# *Lucilia sericata* (Diptera: Calliphoridae) as Agent of Myiasis in a Goose in Italy and a Review of Myiasis by This Species in Birds

**DOI:** 10.3390/insects13060542

**Published:** 2022-06-13

**Authors:** Marco Pezzi, Stjepan Krčmar, Federica Mendicino, Francesco Carlomagno, Domenico Bonelli, Chiara Scapoli, Milvia Chicca, Marilena Leis, Teresa Bonacci

**Affiliations:** 1 Department of Life Sciences and Biotechnology, University of Ferrara, Via L. Borsari 46, 44121 Ferrara, Italy; chiara.scapoli@unife.it (C.S.); milvia.chicca@unife.it (M.C.); marilena.leis@unife.it (M.L.); 2 Department of Biology, Josip Juraj Strossmayer University of Osijek, Cara Hadrijana 8/A, HR-31000 Osijek, Croatia; stjepan@biologija.unios.hr; 3 Department of Biology, Ecology and Earth Sciences, University of Calabria, Via P. Bucci, 87036 Rende, Italy; federica.mendicino@unical.it (F.M.); francesco.carlomagno@unical.it (F.C.); domenico.bonelli@unical.it (D.B.); teresa.bonacci@unical.it (T.B.)

**Keywords:** *Anser anser domesticus*, literature survey, *Lucilia sericata*, Southern Italy, traumatic myiasis

## Abstract

**Simple Summary:**

An unusual case of traumatic myiasis by *Lucilia sericata* (Meigen) (Diptera: Calliphoridae), the first by this species described in a bird in Italy, occurred in a domestic goose, *Anser anser domesticus* L. (Anseriformes: Anatidae), living in a rural area of the region Calabria (Southern Italy). The case is discussed together with an updated and detailed review of worldwide cases of myiasis by *L. sericata* in birds.

**Abstract:**

Myiasis is a type of parasitosis by larvae of Diptera that may affect vertebrates, including wild and domestic birds. Traumatic myiasis was discovered in a domestic goose, *Anser anser domesticus* L. (Anseriformes: Anatidae), in June 2020 in a rural area of the region Calabria (Southern Italy). The myiasis was caused by *Lucilia sericata* (Meigen) (Diptera: Calliphoridae). In Italy, this was the first case of myiasis by *L. sericata* ever described in a bird. It was also the first case of myiasis detected in a goose in Italy. The description of the case is integrated by a discussion on nonhematophagous dipteran larvae causing myiasis in birds and by an updated and detailed review of literature cases of myiasis by *L. sericata* in birds reported worldwide, useful for monitoring and management of dipteran species of medical and veterinary interest.

## 1. Introduction

Myiasis is commonly defined as a form of parasitism exerted on vertebrates (including humans) by larvae of Diptera actively feeding on live or dead host tissue [1]. The relationship between the myiasigenous larva and the host can be classified as accidental (when larvae are not parasitic but rarely may become so), facultative (when larvae may equally develop on living vertebrates and on organic matter) and obligatory (when larvae may develop only on a living host) [2,3,4]. In regard to birds, cases of myiasis have been reported involving species with “hematophagous” and “nonhematophagous” larvae [5]. The species with hematophagous larvae are agents of a type of obligatory myiasis called “sanguinivorous myiasis”, a term indicating larvae of Diptera with ectoparasitic and bloodsucker behaviour [1] found in nests of birds mostly belonging to the order Passeriformes. In this myiasis, the hematophagous larvae express different behaviours, ranging from residing in the nest material and intermittently transferring to nestlings for blood meals to burrowing under the skin to develop and leaving the host to pupate [5]. The species with nonhematophagous larvae are agents of obligatory and facultative myiasis in birds, frequently of the cutaneous type associated with traumatic wounds [5,6,7].

Here we describe a case of traumatic myiasis in a domestic goose, *Anser anser domesticus* L. (Anseriformes: Anatidae), caused by *Lucilia sericata* (Meigen) (Diptera: Calliphoridae) in the region Calabria (Italy), together with an updated and detailed review on worldwide cases of myiasis by *L. sericata* in birds.

## 2. Case Report

**Figure 1:**
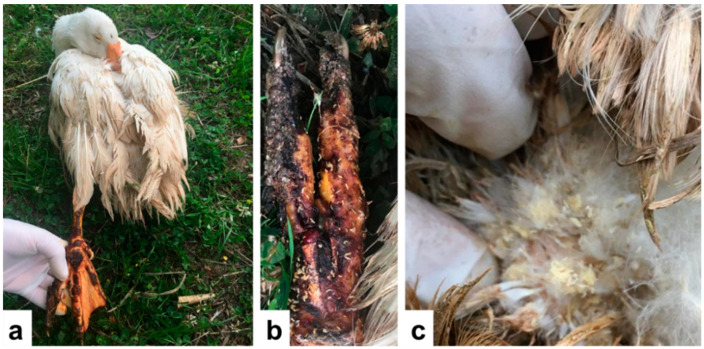
Traumatic myiasis by *Lucilia sericata* in a domestic goose. (**a**) Left leg and foot of the goose, showing trauma by rat bites. (**b**) Dorsal view of the foot, showing wound infested by dipteran larvae. (**c**) Plumage near the cloaca infested by eggs and larvae.

The case involved a 5-year-old privately owned male domestic goose, *Anser anser domesticus* L. (Anseriformes: Anatidae), in Santo Stefano di Rogliano (Cosenza, Calabria, Southern Italy). The goose was affected by leg locomotory problems; therefore, the owners decided to move it from the courtyard to a separate cage in order to avoid attacks from hens. The owners also administered spiramycin (125 mg daily) orally to the goose as a therapy for locomotory problems. When the goose was inside the cage, it was bitten on its left foot by rats, and the owners cleaned and disinfected the wound with povidone-iodine. On 15 June 2020, during an inspection, the owners found several larvae infesting the wound and larvae and eggs infesting the cloacal region (Figure 1). After taking photographs of the infested wound, they removed the larvae with tweezers and stored them alive in an aerated test tube, disinfecting the wound with povidone-iodine. However, the goose died five days after the discovery of larvae.

The total number of living larvae collected was 19. They were brought to the Laboratory of Applied and Forensic Entomology of the Department of Biology, Ecology and Earth Sciences, University of Calabria (Arcavacata di Rende, Cosenza, Italy): 12 of them were rapidly killed by quick immersion in hot water (about 90 °C), and their length in mm was measured (average 3.1 ± 0.3 mm). The 12 larvae were then fixed and stored in 90% ethanol. The remaining seven larvae were all reared to adults in plastic boxes containing about 300 g minced pork liver at 24 ± 0.5 °C, 60% relative humidity and a 12/12 (L/D) photoperiod. Adult flies were exposed to CO_2_ to induce torpor, placed in individual test tubes, painlessly killed by exposure to −20 °C and stored in 80% ethanol. Species identification was carried out based on the morphology of male adults, using a Nikon SMZ 800 stereomicroscope (Nikon Instruments Europe, Amsterdam, The Netherlands) and specific taxonomical keys [8]. The morphological investigation confirmed that the species agent of myiasis was *Lucilia sericata* (Meigen) (Diptera: Calliphoridae).

## 3. Bibliographic Methods

The literature search had no time and language limits, generally following previously described methods [9]. The initial set of publications on myiasis caused by nonhematophagous larvae in birds was obtained by PubMed-indexed literature, and the search was extended through web engines. The keywords used for this investigation were: birds, Diptera infestation, dipteran infestation, domestic bird, facultative myiasis, fly infestation, geese, goose, myiasis, obligatory myiasis and wild bird. When a given publication was not freely available from the web or not available in printed form in university libraries, it was obtained through interlibrary services. The initial set of publications was composed of case reports and general works on myiasis. Among these, there were two books, a review and four book chapters, including data about published cases of myiasis in humans and animals [1,2,4,10,11], myiasis in wildlife in the Nearctic region [12] and several examples of published cases of myiasis in wild birds [5]. This initial set of publications was used as a base for extending the investigation, always consulting the original study, verifying its content and checking the reference list in order to find more cases. In turn, for all publications consulted, the reference list was checked for further cases of myiasis by nonhematophagous larvae in birds in order to obtain a general overview of facultative and obligatory species and possible cases of association of two or more species affecting birds. All data were collected on a spreadsheet programme.

The literature search was simultaneously conducted for cases of myiasis in birds by *L. sericata*. In all sorted publications, the reference list was again checked for further cases of myiasis by *L. sericata* in birds in order to obtain an updated and detailed review. The literature data were collected on another spreadsheet programme, including (when available) the host bird (common name and scientific name), the infestation site, the clinical history of the host, the type of myiasis, the possible association with other Diptera species, the number of cases and the country where the cases were reported. The literature search was conducted until March 2022.

## 4. Results and Discussion

The reported case is the first of myiasis by *L. sericata* ever described in a bird in Italy and also the first case of myiasis involving a goose as a host reported in Italy.

Myiasis in wild and domestic birds is a well-documented type of infestation [1,5,12]. Among Diptera reported as agents of hematophagous myiasis in birds, there are species belonging to the genera *Protocalliphora* (Calliphoridae), *Passeromyia*, *Philornis* (Muscidae) and *Neottiophilum* (Piophilidae) [1,5,13,14,15,16]. These genera typically affect nestling birds and cause severe lesions and mortality, especially in Passeriformes [5]. As in other vertebrate hosts, Diptera causing nonhematophagous myiasis in birds are divided into agents of obligatory and facultative myiasis. Among species causing obligatory myiasis in birds there are *Cochliomyia hominivorax* (Coquerel) [17,18,19] and *Chrysomyia bezziana* Villeneuve (Diptera: Calliphoridae) [20,21], *Wohlfahrtia magnifica* (Schiner) [1,22,23] and *Wohlfahrtia opaca* (Coquillett) (syn. *Wohlfahrtia vigil* (Walker)) (Diptera: Sarcophagidae) [24]. Among the cases of myiasis caused by *W. magnifica*, several were reported in association with *L. sericata* [23]. Some cases of obligatory myiasis by *Dermatobia hominis* Linnaeus Jr. in Pallas (Diptera: Oestridae) were reported in chicken [25]. One case of obligatory myiasis by the genus *Cuterebra* (Diptera: Oestridae) was reported in a male American woodcock, *Scolopax minor* Gmelin (Charadriiformes: Scolopacidae). The larva extracted from the woodcock probably belonged to the species *Cuterebra buccata* (Fabricius) (Diptera: Oestridae) [26].

Among species reported as agents of facultative myiasis in birds there are *Calliphora vicina* Robineau-Desvoidy [27,28,29], *Calliphora augur* (Fabricius) [30], *Cochliomyia macellaria* (Fabricius) [25,31], *Lucilia illustris* Meigen [27], *Lucilia cuprina* (Wiedemann) [32,33], *Lucilia eximia* (Wiedemann) [13,34], *Lucilia richardsi* Collin (Diptera: Calliphoridae) [35], *L. sericata* (see Table 1) and *Sarcodexia lambens* (Wiedemann) (Diptera: Sarcophagidae) [13]. Cases of facultative myiasis by unreported species have also been described, involving the genera *Calliphora* [7,29,36], *Lucilia* [25,29,37] and *Sarcophaga* (Diptera: Sarcophagidae) [38]. In other cases of myiasis in birds, only the dipteran family was reported (Muscidae and Sarcophagidae) [25]. During a survey on the microbiota of hematophagous ectoparasites in migratory birds on the Italian territory, the presence of *Lucilia caesar* (Linnaeus) (Diptera: Calliphoridae) was reported on one individual of the common kestrel, *Falco tinnunculus* Linnaeus (Falconiformes: Falconidae), but the case was not identified as myiasis nor described in any detail [39]. There are also reports of myiasis in birds caused by associations of the above-mentioned species, such as *L. sericata* with *L. cuprina* [32], *L. sericata* with *L. illustris* [27] and *L. sericata* with *W. magnifica* [23]. There is also a report concerning two chicks of *Ramphocelus dimidiatus* Lafresnaye (Passeriformes: Thraupidae) with subcutaneous myiasis caused by the hematophagous species *Philornis glaucinis* Dodge & Aitken (Diptera: Muscidae) and two nonhematophagous and facultative species, *S. lambens* and *L. eximia*. The authors proposed the hypothesis that the infestation by the two nonhematophagous species occurred secondarily due to the wounds caused by *P. glaucinis* [13].

*Lucilia sericata*, a synanthropic species, may develop worldwide on carrions but also on waste and manure [40]. Due to its ability to colonize corpses, including human ones, this species has forensic relevance and is considered an indicator for the estimation of post-mortem interval and other forensic data [41,42,43]. From a medical and veterinary point of view, *L. sericata* is an agent of primary and facultative myiasis, mostly in sheep but also in other wild and domestic animals and humans [44,45]. Literature examples of cases of wild birds affected by nonhematophagous larvae of Diptera, including *L. sericata*, were previously reported [5]. A total of 46 confirmed cases of myiasis in birds by *L. sericata*, including that described in this study, were reported from 1971 [46] to date (Table 1). However, in a veterinary study in captive Falconiformes, myiasis by *L. sericata* was mentioned without reporting the number of cases and other relevant data [7]. In another case reported in Finland in a crane, *Grus grus* (Linnaeus) (Gruiformes: Gruidae), the species was most likely identified as *L. sericata* [47]. Four cases of myiasis in *F. tinnunculus* were reported in Austria as caused by *L. sericata* and *C. vicina*, without mentioning how many of these cases were due to the first and/or the second species [27]. The most numerous cases among the confirmed ones of myiasis by *L. sericata* in birds (19 out of 46) occurred in Hungary because an extensive survey of prevalence and clinical manifestations of traumatic myiasis was conducted on five geese flocks [23]. In this survey, the most frequent body parts affected were the wings. Since *L. sericata* has a worldwide distribution, myiasis in birds by this species has been reported to date in Austria, Finland, Hungary, Iran, Iraq, Israel, Peru and Turkey (Table 1). In Italy, *L. sericata* has been reported as an agent of myiasis since the early 1900s in humans [48,49,50,51,52,53] and in cats [54,55,56], dogs [54,57,58], sheep [54] and a rabbit [59]. Among other myiasigenous species reported in animals in Italy, there are *Oestrus ovis* (Linnaeus) (Diptera: Oestridae) [60], *W. magnifica* [61], *Sarcophaga argyrostoma* (Robineau-Desvoidy) [56], *Sarcophaga portschinskyi* (Rohdendorf) [57], *Sarcophaga tibialis* Macquart (Diptera: Sarcophagidae) [55], *C. vicina* [56,58,62], *Lucilia ampullacea* Villeneuve (Diptera: Calliphoridae) [54], *L. caesar* [63], *L. illustris* [54] and *Muscina stabulans* (Fallén) (Diptera: Muscidae) [57]. Besides the rural hilly area (around 700 a. s. l.) where the present case occurred in Southern Italy, this species has been reported as an agent of myiasis in animals in lowland rural areas of Northern Italy [55,56,58]. Based on the literature data, apparently, this species is an agent of myiasis affecting animals and humans in most of the Italian territory [48,49,50,51,52,53,54,55,56,57,58,59]. The most common type of myiasis caused by *L. sericata* in birds worldwide is cutaneous myiasis, with wounds as a predisposing condition, especially caused by bone fractures due to severe traumas (Table 1). Myiasis by *L. sericata* in birds has also been reported in the oral cavity, eyes, cloaca and phallus. The affected orders included Accipitriformes, Anseriformes, Ciconiiformes, Falconiformes, Galliformes, Gruiformes, Passeriformes and Strigiformes (Table 1). In the described case, three predisposing conditions could have favoured the traumatic myiasis. The first condition is the immobility of the goose, which makes it vulnerable to attacks from other animals, and the second one is a series of bites from rats. The third condition is the accumulation of faeces due to immobility that, together with the open wounds, may have attracted blowflies. The main predisposing conditions reported for the development of myiasis in geese are wounds caused by the plucking of feathers but also bacterial infections of the phallus [23]. In geese, cases of myiasis by *L. sericata* have been reported in Israel [46] and in Hungary, also in association with *W. magnifica* [23]. Two cases of myiasis by *C. hominivorax* in geese were reported in the USA between 1935 and 1936 [17]. *Wohlfahrtia magnifica* was also reported as an agent of myiasis in geese [1], including fourteen cases described in Hungary, of which seven were in association with *L. sericata* [23]. Recently, one case of otomyiasis by *Sarcophaga* spp. was reported [38].

The present one is the first case of myiasis described in Italy involving a goose and provides information about the ability of this dipteran species to attack domestic birds in the Italian territory. This case is also relevant for raising attention in fowl breeders and veterinarians to increase surveillance for prevention of this severe type of parasitosis that may seriously compromise animal welfare.

**Table 1:** Cases of myiasis by *Lucilia sericata* in birds. Abbreviations: *A. anser*, *Anser anser*; *As. flammeus*, *Asio flammeus*; *As. otus*, *Asio otus*; *B. bubo*, *Bubo bubo*; *Bu. rufinus*, *Buteo rufinus*; *C. ciconia*, *Ciconia ciconia*; *Ci. aeruginosus*, *Circus aeruginosus*; *Co. corone*, *Corvus corone*; *F. peregrinus*, *Falco peregrinus*; *F. sparverius*, *Falco sparverius*; *F. tinnunculus*, *Falco tinnunculus*; *G. gallus domesticus*, *Gallus gallus domesticus*; *Gr. grus*, *Grus grus*; *Gy. fulvus*, *Gyps fulvus*; *Pa. unicinctus*, *Parabuteo unicinctus*; *Pe. apivorus*, *Pernis apivorus*; *S. aluco*, *Strix aluco*; UN, unreported. * In association with *Lucilia illustris*; ** seven out of 19 cases in association with *Wohlfahrtia magnifica*; and *** in association with *Lucilia cuprina*.

Order	Genus/Species	Common Name	Infestation Site	History	Type of Myiasis	N. Cases	Country	References
Accipitriformes	*Bu. rufinus*	Long-legged buzzard	Humeral	UN	Traumatic	1	Turkey	[64]
			UN	UN	Traumatic	1	Turkey	[29]
	*Ci. aeruginosus*	Western marsh harrier	Left shoulder	Open fracture of the humerus	Facultative	1	Austria	[6]
			UN	Wound	Facultative	1	Austria	[6]
	*Gy. fulvus*	Griffon vulture	Beak cavity, cloaca and around, and eyes ring	Poor physical conditions	Facultative	1	Austria	[27]
	*Pa. unicinctus*	Harris’s hawks	Cloaca and around	Poor physical conditions	Cloacal	1	Peru	[33]
	*Pe. apivorus*	European honey buzzard	UN	Wound	Facultative	1	Austria	[6]
			UN	UN	Facultative	1 *	Austria	[27]
Anseriformes	*A. anser*	Goose	Back, breast, head, neck, phallus and wing	Wound and infection	Traumatic	19 **	Hungary	[23]
			Wings and caudal area	Poor physical condition and wound	Cutaneous	1	Israel	[46]
			Left leg and cloaca	Poor physical condition and wound by bite	Traumatic	1	Italy	Present report
Ciconiiformes	*C. ciconia*	White stork	Thorax	Wound by bite	Facultative and wound	1	Austria	[65]
Falconiformes	*Falco* spp.	Kestrel	UN	UN	UN	UN	UN	[7]
	*F. peregrinus*	Peregrine falcon	UN	UN	UN	UN	UN	[7]
	*F. tinnunculus*	Common kestrel	Right wing	Wound	Facultative and wound	1	Austria	[65]
			UN	UN	Facultative	UN	Austria	[27]
Galliformes	*G. gallus domesticus*	Rooster	Cloaca and tail	Poor physical conditions	Cloacal	1	Peru	[33]
	*Meleagris* spp.	Turkey	Posterior half of body	Poor physical conditions	Cutaneous	1	Iraq	[66]
Gruiformes	*Gr. grus*	Common crane	Armpit, base of thigh and tail	Poor physical conditions	UN	1	Finland	[47]
Passeriformes	*Co. corone*	Carrion crow	Abdomen	UN	Traumatic	1	Turkey	[64]
Strigiformes	*As. flammeus*	Short-eared owl	Right wing	Open fracture of wing	Facultative and wound	1	Austria	[67]
	*As. otus*	Long-eared owl	UN	UN	Facultative	1	Austria	[27]
	*B. bubo*	Eurasian eagle-owl	UN	Wound	Facultative	2	Austria	[6]
			Right paw	Wound	Facultative	1	Austria	[27]
			UN	UN	Facultative	1	Austria	[27]
			Right wing	Wound	Cutaneous	1 ***	Iran	[32]
	*S. aluco*	Tawny owl	UN	Wound	Facultative	1	Austria	[6]
			UN	UN	Facultative	2	Austria	[27]
	Unidentified	Owl	Right wing	Wound	Traumatic	1	Turkey	[68]

## Data Availability

The data supporting this investigation are available from the corresponding author Marco Pezzi upon reasonable request.
